# *Toxoplasma gondii*-derived antigen modifies tumor microenvironment of Ehrlich solid carcinoma murine model and enhances immunotherapeutic activity of cyclophosphamide

**DOI:** 10.1007/s12032-023-01994-y

**Published:** 2023-04-04

**Authors:** Cherine A. Ismail, Maha M. Eissa, Maha R. Gaafar, Layla K. Younis, Nahla El Skhawy

**Affiliations:** 1 Department of Clinical Pharmacology, Faculty of Medicine, Alexandria University, Alexandria, Egypt; 2 Department of Medical Parasitology, Faculty of Medicine, Alexandria University, Alexandria, Egypt; 3 Department of Pathology, Faculty of Medicine, Alexandria University, Alexandria, Egypt

**Keywords:** Autoclaved *Toxoplasma* vaccine, Ehrlich carcinoma, Immunotherapy, Cyclophosphamide, CD8^+^ T/Treg, VEGF

## Abstract

Pathogen-based cancer vaccine is a promising immunotherapeutic weapon to stimulate cancer immunosuppressive state. *Toxoplasma gondii* is a potent immunostimulant, and low-dose infection was linked to cancer resistance. Our goal was to evaluate the therapeutic antineoplastic activity of autoclaved *Toxoplasma* vaccine (ATV) against Ehrlich solid carcinoma (ESC) in mice in reference to and in combination with low-dose cyclophosphamide (CP), a cancer immunomodulator. Mice inoculation with ESC was followed by applying different treatment modalities including ATV, CP, and CP/ATV. We evaluated the impact of the different treatments on liver enzymes and pathology, tumor weight, volume, and histopathological changes. Using immunohistochemistry, we evaluated CD8^+^ T cell, FOXP3^+^ Treg, CD8^+^/Treg outside and inside ESC, and angiogenesis. Results showed significant tumor weights and volumes reduction with all treatments with 13.3% inhibition of tumor development upon combined CP/ATV use. Significant necrosis and fibrosis were noted in ESC by all treatments with improved hepatic functions versus non-treated control. Although ATV was almost equivalent to CP in tumor gross and histopathology, it promoted an immunostimulatory activity with significant Treg cells depletion outside ESC and CD8^+^ T cells infiltration inside ESC with higher CD8^+^ T/Treg ratio inside ESC superior to CP. Combined with CP, ATV exhibited significant synergistic immunotherapeutic and antiangiogenic action compared to either treatment alone with significant Kupffer cells hyperplasia and hypertrophy. Exclusively, therapeutic antineoplastic and antiangiogenic activity of ATV against ESC was verified that boosted CP immunomodulatory action which highlights a novel biological cancer immunotherapeutic vaccine candidate.

## Introduction

Parasites are double-edged sword, with a noticeable negative impact on their hosts, while possessing a powerful advantageous immunomodulatory effect that can be exploited for the host’s benefit [1, 2]. This immunomodulatory activity was verified against various immune-related diseases as allergies, autoimmune diseases, and others [3, 4]. Cancer is an immune-related disease with obvious immunosuppression [5]. A powerful T-helper 1 (TH1) immune response induced by some parasites as *Toxoplasma gondii* (*T. gondii*) can be employed to counteract cancer TH2 immunosuppressive dominance [1].

Pathogen-based cancer immunotherapy is an axial tool to counteract cancer immunosuppressive dominance. Bacille Calmette–Guerin (BCG), a *Mycobacterium bovis* live*-*attenuated vaccine, is a cancer bladder therapeutic vaccine [6]. Number of pathogens are now in the pipeline investigating their antineoplastic effectiveness [1, 7].

Relying on data affirming that low-dose chronic asymptomatic *T. gondii* infection provoked an antineoplastic effect [7] and that the low titer anti-*Toxoplasma* antibody is associated with cancer resistance [8] enrich repositioning of *T. gondii* as a vaccine candidate for cancer immunotherapy. Whereas acquiring infection appears non-realistic to seize the antineoplastic activity, parasitic vaccines can be exploited to pursuit this activity.

Autoclaved parasitic vaccines are special type of killed vaccines retaining the essential parasitic immunogenic components [9] that proved to be safe, easy to prepare, stable, and cheap [10]. They revealed high homologous protective immunity against corresponding infection as toxoplasmosis, schistosomiasis, and trichinellosis [9, 11, 12]. Autoclaved cercarial vaccine was protective against experimental schistosomiasis and provoked therapeutic antineoplastic activity against experimental cancer colon in mice [11, 13]. In experimental toxoplasmosis, autoclaved *Toxoplasma* vaccine (ATV) reduced hepatic and splenic load of *T. gondii* tachyzoites, superior to *T. gondii* lysate antigen with rise in splenic CD8^+^ T cells [9]. Since, intratumorally injected attenuated *T. gondii* tachyzoites provoked an antineoplastic role against melanoma model [14], exploring ATV therapeutic antineoplastic activity may promote its enrollment to parasite-based cancer vaccines for immunotherapy.

Cyclophosphamide (CP) has a differential dose-dependent action, an immunosuppressive and immunomodulatory role. Its precise immunomodulatory mechanism is not entirely clear, yet studies suggested a role for selective T regulatory (Treg) cell depletion [15, 16, 17]. Its unique low-dose immunomodulatory action modifies the immunosuppressive tumor microenvironment, which augments the response to main adjunctive therapies while minimizing risk for adverse drug reactions [17]. Since immune-mediated therapies are becoming prevalent in cancer, we investigated ATV therapeutic antineoplastic activity in reference to and in combination with low-dose CP in Ehrlich solid carcinoma (ESC), a well-established murine cancer model.

## Material and methods

### *Toxoplasma gondii* maintenance and vaccine preparation

Live tachyzoites of *T. gondii* (virulent RH HXGPRT (-) strain) were maintained via serial intraperitoneal (*ip*) passages in Swiss albino mice. Harvested tachyzoites were used in animal infection and vaccine preparation [9]. Autoclaved *Toxoplasma* vaccine was prepared as previously described [9, 18]. Collected tachyzoites, from the peritoneal fluid of infected mice, were centrifuged for five minutes (min) at 500×*g* to allow sedimentation of leukocytes and heavier particles. The supernatant was then collected and recentrifuged for five min at 2000×*g*. The supernatant was discarded, and the sediment was suspended in phosphate-buffered saline (PBS) and washed three times for five min at 2000×*g*. The final pellet containing tachyzoites was resuspended in PBS then autoclaved at 120 °C, under pressure of 15 lb for 15 min. Then, they were kept at − 20 until being lyophilized for later use. Quantification of protein concentration of autoclaved *Toxoplasma* vaccine was performed using the NanoDrop™ 2000 spectrophotometer (Thermoscientific) at an absorption wavelength of 280 nm and proteins concentration were expressed in mg/ml [19].

### Ehrlich ascites carcinoma maintenance

Ehrlich ascites carcinoma cells (EAC) in mouse were obtained from the National Cancer Institute (Cairo, Egypt). EAC cells were maintained via serial *ip* passages of 0.2 ml of diluted EAC containing 2.5 × 10^6^ EAC cells in female Swiss albino mice [20].

### Animals

Sixty-six female Swiss albino mice, 5–6 weeks old (20–25 g) were assigned to this study. Mice were obtained from the animal house, Department of Medical Parasitology, Faculty of Medicine, Alexandria University. They were housed under standard laboratory conditions (27 ± 2 °C; 70–80% humidity; 12-h light/dark cycle) with standard pellet diet and water ad libitum. Mice were handled in accordance to the ARRIVE guidelines for animal care and in compliance to the Institutional Animal Care and Use Committee in Faculty of Medicine, Alexandria University (IACUC, 0201396).

### Experimental design

#### Ehrlich solid carcinoma induction

For ESC induction, 0.2 ml of diluted EAC containing 2.5 × 10^6^ cells was injected subcutaneously on the back of each mouse [20]. Efforts were made to reduce animal suffering through daily observation and recording of pre-set humane endpoints including lethargy, abnormal mobility, weight reduction, labored breathing, or diarrhea. Any mouse showed any humane endpoint was immediately euthanized, excluded, and replaced.

#### Experimental groups

Sixty mice were inoculated with EAC for ESC induction as mentioned above, whereas the remaining six served as normal control. Six days post-ESC induction, mice were randomly divided into two main groups: ESC control (15 mice) and ESC-treated groups (45 mice). ESC-treated groups were randomly subdivided into 3 equal subgroups (15 mice each) based on the treating agent and included CP-treated mice, treated with two doses of CP 50 mg/kg (Endoxan Baxter^®^) *ip* two weeks apart starting on the 6th day post-EAC inoculation [16], ATV-treated mice, treated with two doses of 25 μg ATV [21] intradermally over the sternum two weeks apart starting on the 7th day post-EAC inoculation [22], and combination subgroup CP/ATV-treated mice, obtained CP and ATV with the same dosage regimen of the previous two subgroups. Since CP continuous daily administration may lead to drug resistance and impaired immunomodulation, an intermittent schedule of low-dose CP was adopted to assist its inhibitory effect on both Treg cells number and functions [23, 24]. On day 30 post-ESC induction, mice were anesthetized with *ip* thiopental sodium (45 mg/kg) and blood was collected for biochemical analysis [22]. After euthanizing by an overdose of thiopental, solid tumors and livers were carefully excised.

#### Hepatic transaminases

To assess hepatic functions in untreated and treated ESC mice, liver transaminase enzymes were measured. Chemical auto-analyzer Dimension RxL Max (Siemens Health Care Diagnostics, USA) was used to measure Aspartate aminotransferase (AST) and Alanine aminotransferase (ALT).

#### Pathological examination

Both liver and ESC were carefully excised from each mouse. Livers were fixed in 10% buffered formalin and processed for histopathological examination by H&E stain to assess pathological changes. Hepatic histopathological changes were evaluated using a semiquantitative scoring system, as described before with some modification [25]. All excised ESC tumors were weighted, and tumor volumes were calculated as follows: 1/2 × *L* × *W* × *H.* Here, L, W, and H are the length, width, and height of each tumor, respectively, and expressed in mm^3^ [26]. All tumor samples were fixed in 10% buffered formalin, processed for histopathological examination by H&E staining, and Masson trichrome to assess the degree of fibrosis. Evaluation of the stained sections to assess degree of fibrosis and necrosis was performed using a semiquantitative grading system, as described previously with some modifications [27, 28]. Histopathological examination was performed in a blind manner.

#### Immunohistochemistry

For immunohistochemical (IHC) evaluation, all ESC sections were subsequently stained for CD8^+^ T cells, Forkhead box P3^+^ (FOXP3^+^) regulatory T cells (Treg), and vascular endothelial growth factor (VEGF) using horseradish peroxidase (HRP) (UltraVision ONE HRP Polymer, Thermoscientific). These antibodies were used according to the manufacturer’s guidance: Anti-CD8 (Ab-1) monoclonal antibody (Thermoscientific), Anti-FOXP3 (86 D) monoclonal antibody (BioCare Medical), and Anti-VEGF polyclonal antibody (BioGenex). Sections were deparaffinized and stained for IHC as previously described [22]. For each IHC run, and for each antibody, a positive and negative control was included. Negative controls were included by omission of the primary antibody. For FOXP3 and CD8, sections of tonsils or lymph nodes were used as positive control while for VEGF, angiosarcoma sections were used. Slides were photographed, analyzed, and expressed as the mean number of positively stained cells/HPF [13, 22].

### Statistical analysis

Statistical analysis was performed using IBM^®^ SPSS^®^ Statistics, version 25. Quantitative data were analyzed using one-way analysis of variance (ANOVA) with post hoc Tukey’s test for comparison between groups. Qualitative data were analyzed with Kruskal–Wallis test, and significance were adjusted by Bonferroni correction for multiple tests. Data presented are the average of two experimental replicates and expressed as mean ± standard error of the mean (SEM). Significance was considered when *p* values were ˂ 0.05.

## Results

### Hepatic functions and histopathological changes

#### Hepatic transaminases

**Fig. 1:**
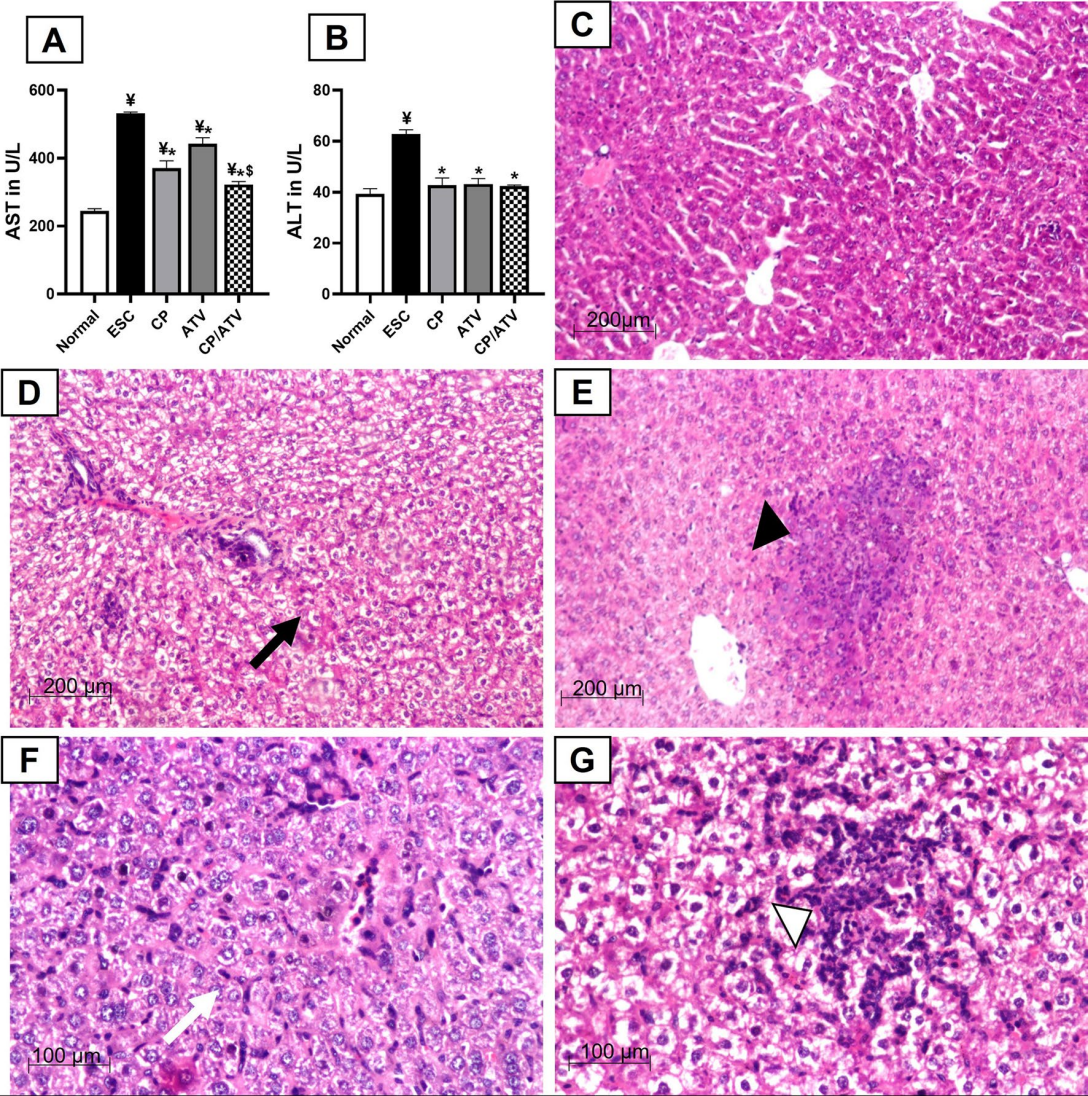
Hepatic transaminases and representative photomicrographs of H&E-stained liver sections: mean level of serum AST and ALT in **A, B**, respectively. ¥*p* < 0.05 versus normal control; *p < 0.05 versus ESC control; $*p* < 0.05 versus ATV-treated group. AST: Aspartate aminotransferase; ALT: Alanine aminotransferase; Normal: normal control; ESC: Ehrlich solid carcinoma control group; CP: cyclophosphamide-treated ESC group; ATV: Autoclaved *Toxoplasma* vaccine*-*treated ESC group; CP/ATV: Combined cyclophosphamide-treated and ATV-treated ESC group. Hepatic H&E sections showed: **C** normal preserved architecture in normal control. **D** Diffuse fatty changes (black arrow) with mild inflammation in ESC control. **E** Focal fatty changes and scattered granuloma composed of inflammatory cells (black arrowhead) in CP-treated group. **F** Moderate inflammatory cells aggregate mainly lymphocytes especially in the sinusoidal spaces with Kupffer cell hypertrophy (white arrow) in ATV-treated group. **G** Mild to moderate degree of portal inflammation with foci of lymphocytic aggregates and focal necrosis, marked hypertrophy and hyperplasia of Kupffer cells and sinusoidal lymphocytic infiltration (white arrowhead) in CP/ATV-treated group

As shown in Fig. 1A, B, liver enzymes (AST and ALT) of ESC control were significantly elevated compared to normal control (*p* < 0.05). Combined CP/ATV and each treatment alone significantly reduced liver enzymes compared to ESC control (*p* < 0.05). Within treated groups, treatment with CP alone or CP/ATV significantly reduced AST level compared to ATV sole treatment (*p* < 0.05). However, for ALT, no significance difference was reported between CP, and CP/ATV versus ATV sole treatment.

#### Hepatic histopathological assessment

In normal control, liver H&E sections displayed preserved hepatic architecture, while ESC control showed mild periportal inflammatory infiltrates with diffuse fatty changes (Fig. 1C, D, respectively). Liver sections from CP-treated mice showed mild central necrotic areas with mild inflammatory cells infiltrates in addition to focal fatty changes with scattered granuloma of epithelioid and inflammatory cells (Fig. 1E). However, liver sections from ATV-treated mice showed moderate inflammatory cells aggregates mainly lymphocytes, especially in the sinusoidal spaces with Kupffer cell hypertrophy (Fig. 1F). Lastly, CP/ATV treatment revealed mild to moderate portal inflammation with foci of lymphocytic aggregates and focal necrosis. Marked hypertrophy and hyperplasia of Kupffer cells, and sinusoidal lymphocytic infiltration were also noted (Fig. 1G).

### Ehrlich solid carcinoma examination

#### ESC gross pathological examination (weight and volume)

**Fig. 2:**
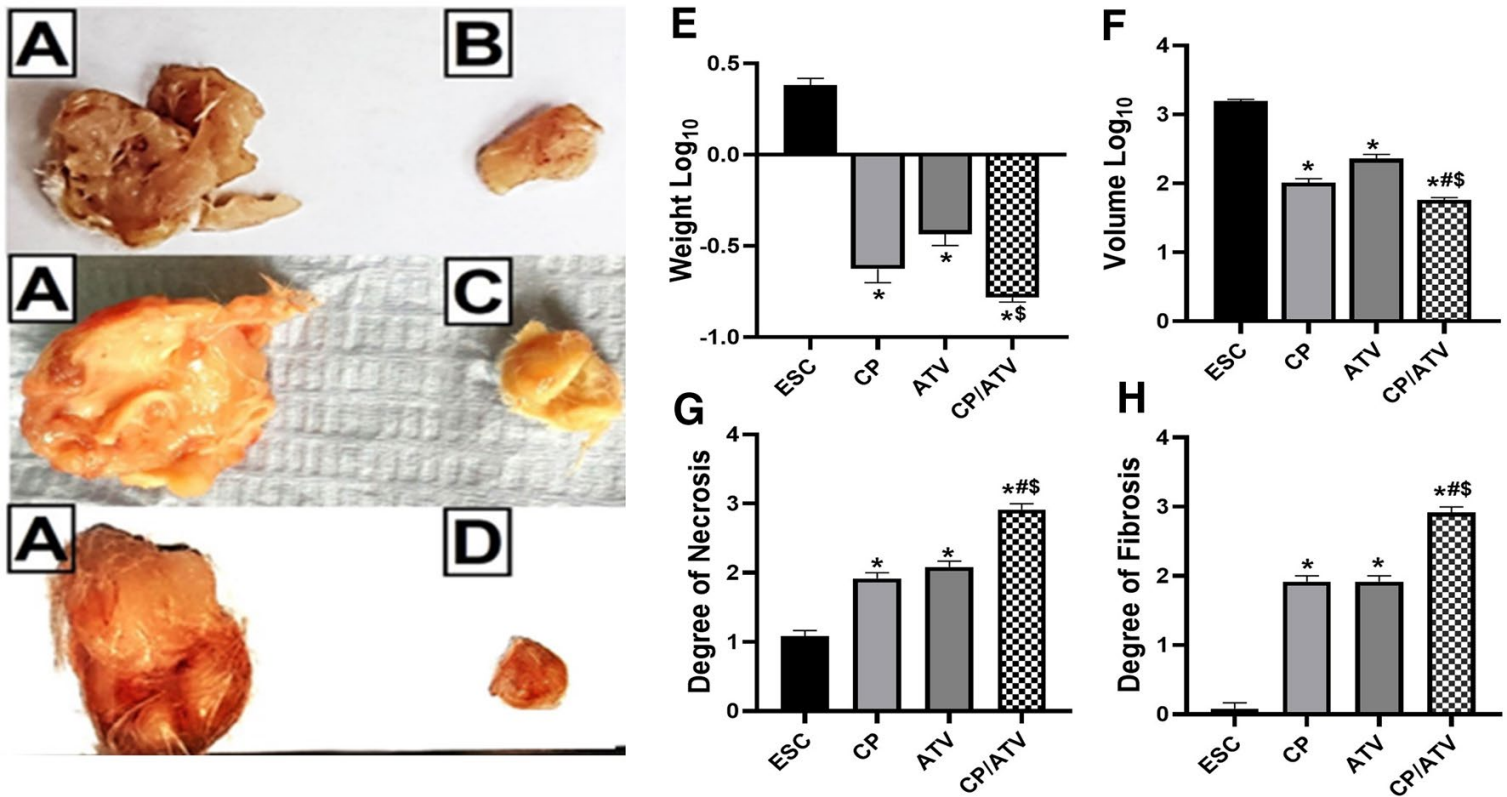
**A**–**D** Representative gross pathological pictures of Ehrlich solid carcinoma. **A** ESC from ESC control group. **B** ESC from CP-treated group. **C** ESC from ATV-treated group. **D** ESC from CP/ATV-treated group. **E–H** Characteristics of Ehrlich solid carcinoma showing: ESC weight and volume Log_10_ in **E, F**, respectively. Histopathological degree of necrosis and fibrosis are shown in **G**, **H**, respectively. * *p* < 0.05 versus ESC control; #*p* < 0.05 versus CP-treated group; $*p* < 0.05 versus ATV-treated group. ESC: Ehrlich solid carcinoma; CP: cyclophosphamide; ATV: Autoclaved *Toxoplasma* vaccine. CP/ATV: combined cyclophosphamide and ATV-treated ESC group

Pictures of the tumors gross pathology from ESC control, CP-treated, ATV-treated, and CP/ATV-treated groups are shown in Fig. 2A–D, respectively. Only with combined CP/ATV treatment, 13/15 mice developed ESC with an inhibition rate of 13.3%. Upon comparing Log_10_ of ESC weight and volume, results showed that all treatments significantly reduced ESC weight and volume compared to ESC control (*p* < 0.05) with the highest reduction achieved with CP/ATV in both parameters (Fig. 2E, F, respectively). Within treated groups, no significance difference was reported in ESC Log_10_ weight between CP, ATV, while CP/ATV significantly reduced ESC weight versus ATV sole treatment (*p* < 0.05). Additionally, CP/ATV significantly reduced ESC volume compared to either treatment alone, and CP treatment was superior to ATV alone in volume reduction (*p* < 0.05).

#### ESC histopathological examination

##### ESC H&E and Masson trichrome staining

**Fig. 3:**
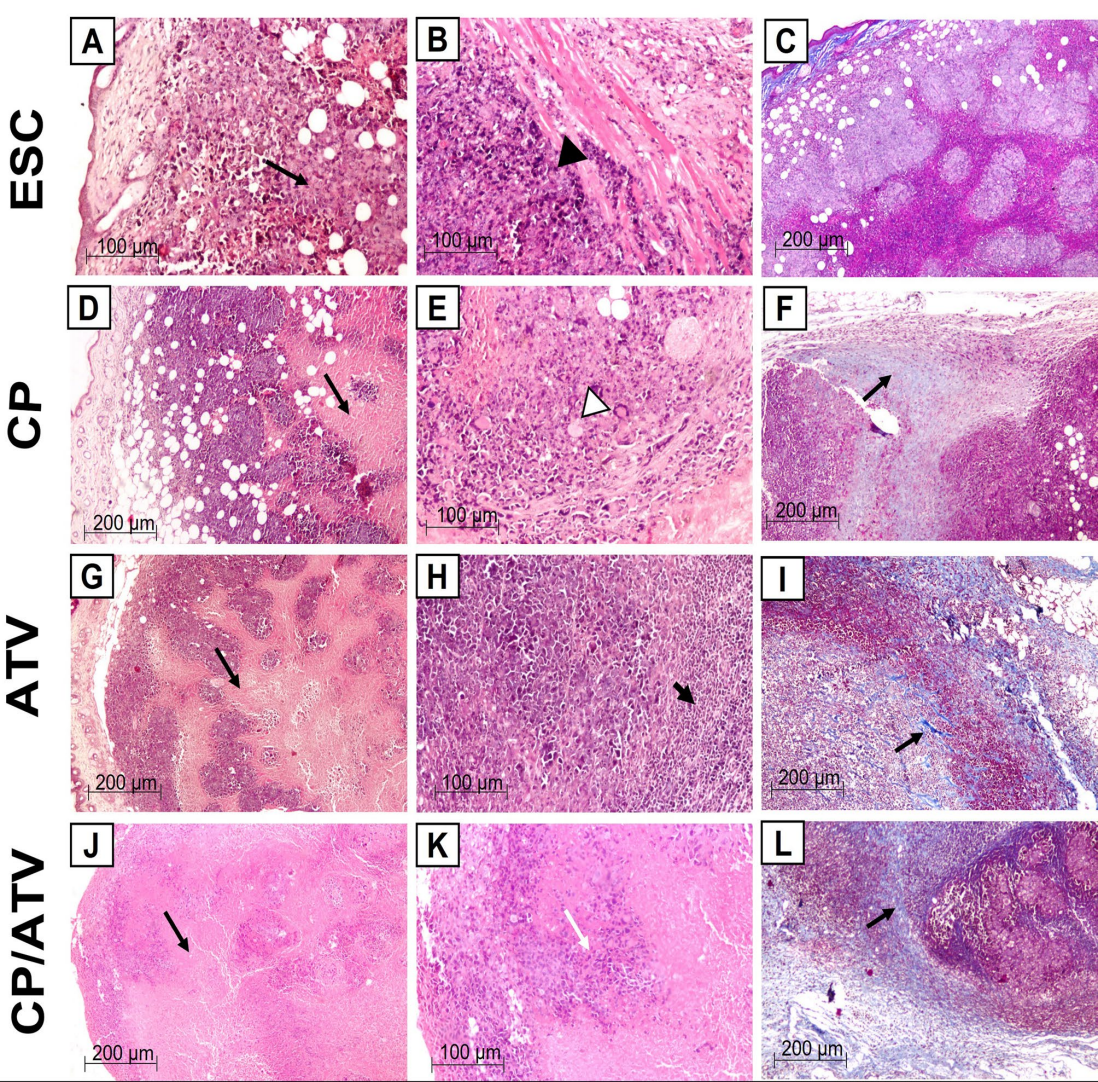
Representative photomicrographs of H & E and Masson trichrome-stained Ehrlich solid carcinoma sections.** A–C** Sections from ESC control showing; **A** shapes of tumor cells invading subcutaneous fat with foci of necrotic areas (grade 1+) (black arrow), and **B** tumor cells invading underlying muscle (black arrowhead) (H&E).** C** Masson trichrome-stained ESC control sections showing no fibrosis (Masson trichrome). **D–F** Sections of ESC from CP-treated group showing; **D** grade 2 + central necrosis (black arrow) with few viable tumor cells, and **E** malignant cells with bizarre nuclei and giant cell infiltration (white arrowhead) (H&E). **F** Masson trichrome-stained ESC sections from CP-treated sections showing grade 2 + fibrosis. Black arrow points at fibrosis (Masson trichrome). **G–I** Sections of ESC from ATV-treated group showing; **G** grade 2 + central necrosis (black arrow), and **H** remarkable lymphocytic infiltrates (thick black arrow) (H&E). **I** Masson trichrome-stained ESC sections from ATV-treated sections showing grade 2 + fibrosis. Black arrow points at fibrosis (Masson trichrome). **J–L** Sections of ESC from CP/ATV-treated group showing; **J** extensive grade 3 + necrosis (black arrow), and **K** minimal tumor cells (white arrow) (H&E). **L** Masson trichrome-stained ESC sections from CP/ATV-treated sections showing severe grade 3 + fibrosis. Black arrow points at fibrosis (Masson trichrome). ESC: Ehrlich solid carcinoma; CP: cyclophosphamide; ATV: Autoclaved *Toxoplasma* vaccine*.* CP/ATV: combined cyclophosphamide and ATV-treated ESC group

H&E-stained tumor sections in ESC control showed subcutaneous sheets of highly malignant cells with increased nucleocytoplasmic ratio, pleomorphic hyperchromatic nuclei, and numerous mitotic figures, enclosing foci of central necrotic areas (grade 1+) (Fig. 2G). Additionally, some samples showed invasion of the underlying muscle by tumor cells (Fig. 3A, B). Upon treatment with CP, tumor sections showed significant increase in the central necrotic area (grade 2+) compared to ESC control (*p* < 0.05, Fig. 2G) that markedly encroached on the tumor leaving a peripheral rim of malignant cells infiltrated with inflammatory and giant cells (Fig. 3D, E). Similarly, upon treatment with ATV, tumors demonstrated a significant expansion in the central necrotic area (grade 2 +) compared to ESC control (*p* < 0.05, Fig. 2G) and a remarkable decrease in tumor cells with increase in lymphocytic infiltrates surrounding sheets of tumor cells (Fig. 3G, H). Whereas CP/ATV treatment significantly exhibited an extensive necrosis (grade 3+) compared to all groups (*p* < 0.05, Fig. 2G) with minimal tumor cells surrounded by excess of lymphocytic infiltrates (Fig. 3J, K).

Comparing the degree of fibrosis in ESC sections (Fig. 2H), Masson trichrome stain of ESC revealed minimal degree of fibrosis (Nil) in ESC control (grade 0) that increased significantly (grade 2+) in both CP and ATV treatment alone compared to ESC control (*p* < 0.05). Whereas, in CP/ATV treatment, ESC exhibited severe degree of fibrosis (grade 3+) that was even significantly higher compared to both CP-treated and ATV-treated groups (*p* < 0.05, Figs. 2H, 3C, F, I, L, respectively).

##### ESC immunohistochemical analysis

**Fig. 4:**
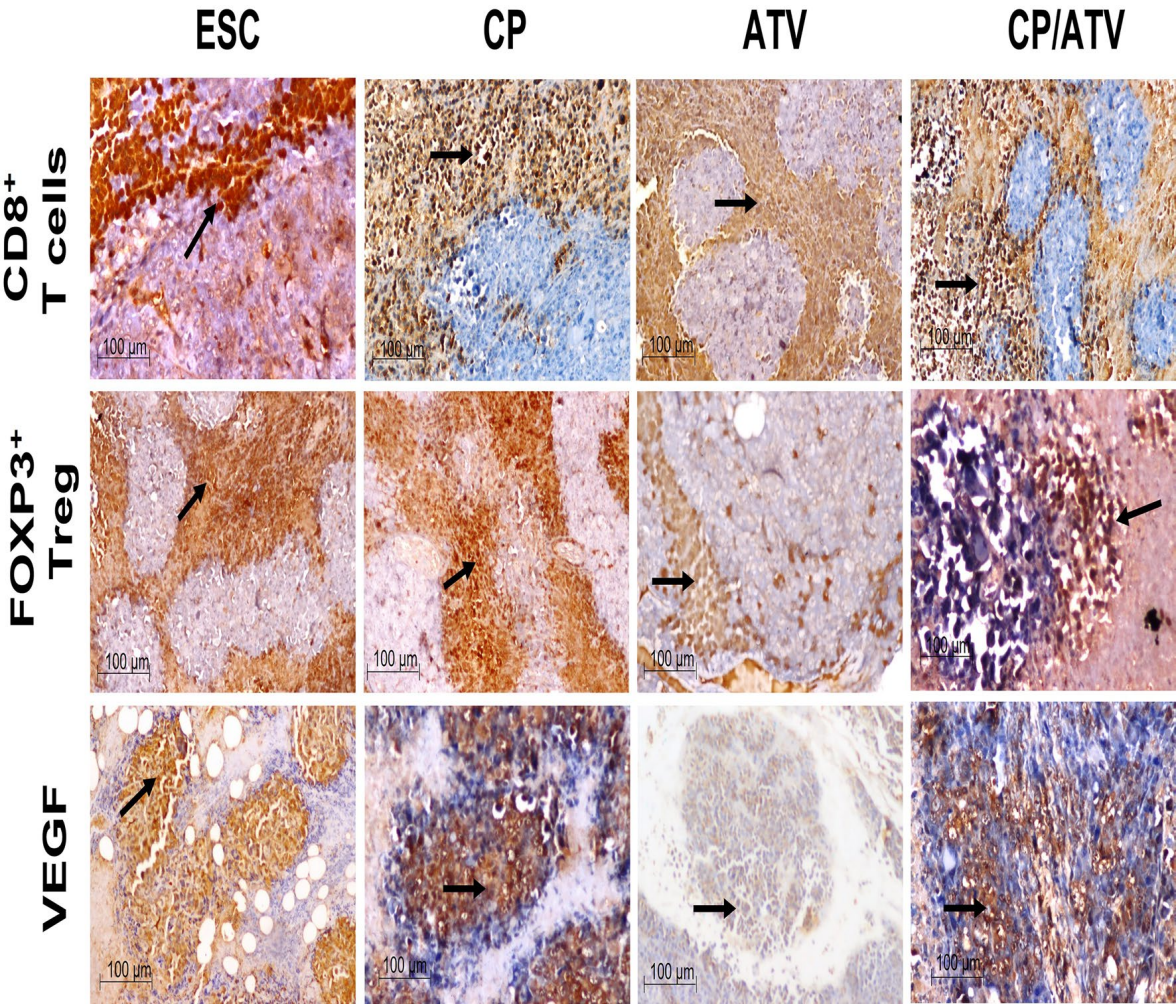
Representative photomicrographs of IHC-stained immune cells and VEGF expression in Ehrlich solid carcinoma. Sections of ESC from CP-treated, ATV-treated, and CP/ATV-treated groups showed abundant CD8^+^ T cells, fewer FOXP3^+^ Treg surrounding and infiltrating tumor tissue and less VEGF expression compared to those of ESC control sections. Black arrow points at positively stained immune cells and VEGF. ESC: Ehrlich solid carcinoma control group; CP: cyclophosphamide-treated ESC group; ATV: Autoclaved *Toxoplasma* vaccine-treated ESC group; CP/ATV: Combined cyclophosphamide-treated and ATV-treated ESC group; VEGF: Vascular endothelial growth factor

**Fig. 5:**
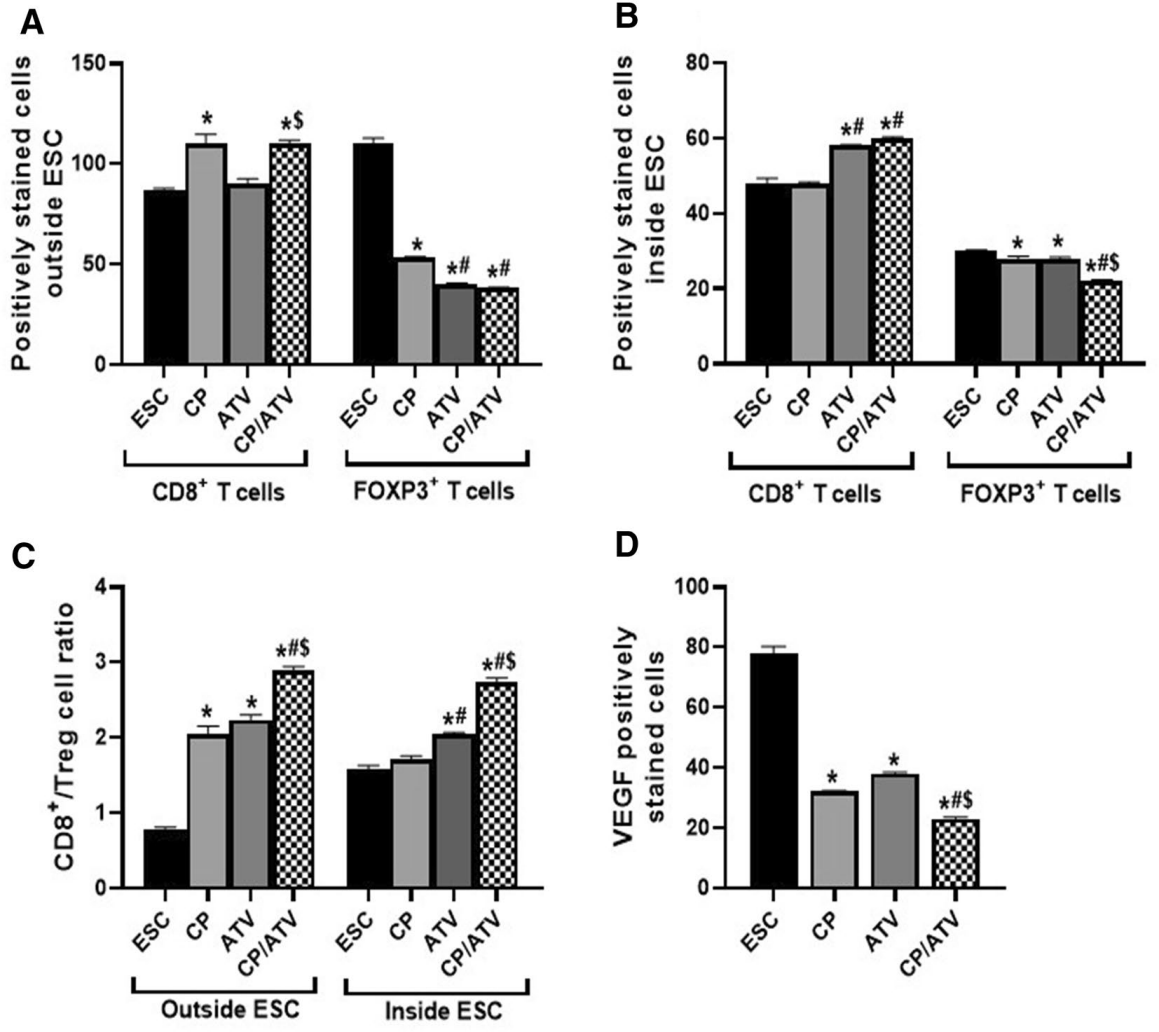
Graphs of the IHC positively stained immune cells (CD8^+^ T and FOXP3^+^ Treg cells) and VEGF expression, showing; average number of CD8^+^ T and FOXP3^+^ Treg cells outside and inside ESC in **A**, **B**, respectively. CD8^+^/Treg cell ratio outside and inside ESC are shown in **C**. Expression of VEGF in ESC is shown in **D**. *p < 0.05 versus ESC control; #*p* < 0.05 versus CP-treated group; $*p* < 0.05 versus ATV-treated group. ESC: Ehrlich solid carcinoma control group; CP: cyclophosphamide-treated ESC group; ATV: Autoclaved *Toxoplasma* vaccine-treated ESC group; CP/ATV: Combined cyclophosphamide-treated and ATV-treated ESC group; VEGF: Vascular endothelial growth factor

*CD8*^+^ *T and Treg cells counts and ratio surrounding and infiltrating ESC*. The IHC of CD8^+^ T and FOXP3 Treg cells surrounding and infiltrating ESC and the statistical analysis of their counts and ratio are shown in Figs. 4 and 5A–C, respectively. Regarding immune cells surrounding ESC, only CP and CP/ATV treatments revealed a significant increase in CD8^+^ T cells versus ESC control (*p* < 0.05). With respect to Treg cells, all treatments promoted significant reduction in Treg cells compared to ESC control, yet their counts in both ATV and CP/ATV treatments were significantly reduced versus that of CP (*p* < 0.05) (Fig. 5A). For immune cells infiltrating ESC, CD8^+^ T cells were significantly increased in both ATV-treated and CP/ATV-treated mice versus ESC control and CP treatment (*p* < 0.05). With respect to Treg cells, all treatments induced significant reduction in cell count compared to ESC control (*p* < 0.05). Treatment with CP/ATV induced significant reduction in Treg cells compared to either treatment alone (*p* < 0.05) (Fig. 5B). To emphasize these findings, CD8^+^/Treg cells ratio was measured, being a crucial prognostic marker anticipating the outcome [29]. In cells surrounding ESC, CD8^+^/Treg cells ratio was significantly higher in all treatments compared to ESC control (*p* < 0.05). Within the treated groups, a significantly higher ratio was documented with CP/ATV, yet no significance difference was recorded between either treatment alone. However, inside ESC, both ATV and CP/ATV treatments exhibited significantly higher CD8^+^/Treg cells ratio versus ESC control and CP treatment (*p* < 0.05). Interestingly, CP/ATV treatment exhibited a significantly higher ratio versus ATV sole treatment (*p* < 0.05) (Fig. 5C).

*ESC vascular endothelial growth factor*. Regarding angiogenesis in ESC, all treated groups revealed significant reduction in VEGF compared to ESC control (*p* < 0.05). The highest reduction in VEGF was achieved by CP/ATV treatment that was significantly less compared to either treatment alone (*p* < 0.05). (Figs. 4, 5D).

## Discussion

The dominance of cancer immunosuppression remarks the significant role of cancer immunotherapy. Aside from the immunomodulatory agents in cancer pipeline, pathogens have evolved as promising candidates. Moreover, low titer of *T. gondii* antibodies was related to cancer resistance [8] and anti-*Toxoplasma* antibodies selectively attached to mouse cancer cell lines [30]. These data justify the investigation of the antineoplastic potential of *Toxoplasma*-derived vaccine.

In this study, induction of ESC in mice universally augmented liver enzymes and impaired hepatic structure evidenced by the observed diffuse fatty changes of hepatocytes compared to normal control, in line with the previous studies [20]. In fact, impacting liver enzymes and architecture has been a confirmed criteria in almost all cancer models [22, 31]. These hepatic deleterious changes induced by ESC were generally corrected by all adopted treatments denoting a positive influence of CP and ATV on hepatic functions being highest with CP/ ATV. This was evidenced by the significant improvement of hepatic enzymes by all treatments. However, from the histopathological background, a generalized hepatic hyperimmune state was detected in the treated mice, particularly in the ATV-treated and CP/ATV-treated group, evidenced by marked hypertrophy and hyperplasia of Kupffer cells, and sinusoidal lymphocytic infiltration. This could speculate an exceptional immune-mediated role of ATV.

It is worth to note that Kupffer cells are liver macrophages resident with antitumor and antimetastatic activity through interferon gamma, interleukin-12, and other inflammatory mediators production that have cytotoxic effect on cancer cells [32]. Moreover, these mediators activate hepatic lymphocytes that migrate to cancerous tissues to interfere with their growth [33]. Normally Kupffer cells can sample tumor cells, yet their efficacy to control tumor growth is limited and cancer immunotherapy can additively enhance Kupffer cell function [34]. These data justify the speculated potent immune-mediated antineoplastic activity of ATV, since fascinating hyperplasia and hypertrophy of Kupffer cells were only detected upon ATV and CP/ATV treatment. However, immune-mediated hepatitis has been reported in patients with solid tumors receiving immunotherapy, while lacking signs of blood hepatotoxicity. This explains our findings of inflammatory hepatic reaction with all adopted treatments that was associated with improvement of hepatic transaminases [35].

These findings match with the previous studies reporting mild elevation of liver transaminases induced by CP standard doses [36], yet, this does not usually coincide with hepatic histopathological changes, as aforementioned [35]. Indeed, treatment with CP showed mild central necrotic areas in liver sections that could be probably caused by CP hepatic metabolism [15]. On the contrary, no hepatic focal necrotic areas were detected upon ATV, denoting a tolerable hepatic impact of ATV as previously reported [9, 18].

Gross pathological examination of ESC excised from mice treated with all adopted treatments revealed significant reduction in both ESC weight and volume compared to ESC control with the highest reduction encountered in CP/ATV-treated mice. This was similarly encountered in the previous studies upon usage of *T. gondii* in treatment of melanoma [14], fibrosarcoma, and sarcoma in animal models [26, 37]. Additionally, the combined CP/ATV inhibited ESC development by 13.3% denoting a synergistic antineoplastic potential of ATV while added to CP. This synergistic effect was more evident in ESC volume compared to both CP and ATV sole treatment. This is probably due to the significant difference in the degree of fibrosis encountered upon CP/ATV treatment compared to that in CP and ATV individual groups. In CP/ATV-treated ESC, excessive fibrosis was probably responsible for the detected markedly shrunken tumor volume.

Histopathological analysis of tumor sections from ESC control disclosed sheets of malignant cells synchronizing with other studies [20]. Foci of central necrosis were noted probably due to hypoxia and nutrient deficiency [38]. While the impact of necrosis on tumor prognosis is query, tumor-induced central necrosis is usually associated with bad prognosis as reported in gastrointestinal and liver tumors [39, 40]. This negative impact is explained by the release of proinflammatory mediators promoting chronic inflammation, which invites immune cells including neutrophils that promote angiogenesis, tumor cell proliferation, and immunosuppression within the tumor [38, 41].

On the contrary, tumor necrosis induced by treatment, chemotherapy, or immunotherapeutic agents as checkpoint inhibitors was correlated with better prognosis through decreasing viable tumor content. The released necrotic cell contents stimulate the immune system, promoting antigen presentation and cytotoxic T cell activity [42, 43]. This fits within our results since all adopted treatments induced significantly more necrosis compared to ESC control. A cumulative effect of CP/ATV treatment promoted extensive necrosis in tumor cells compared to CP and ATV treatment alone. This synergistic effect of CP/ATV treatment indicates a better prognosis since more necrosis denotes better treatment response [43].

Primarily, this necrosis could be due to the immunostimulatory activity of all treatments evidenced by the remarkable increase in lymphocytic aggregates around ESC and giant cell infiltrates by ATV and CP treatment, respectively, that was supported by the current IHC results. Also, necrosis could be due to blood supply deprivation of the ESC that was later justified by diminished VEGF expression. These results can correlate with the potential use of *T. gondii* as checkpoint inhibitors after confirming inhibition of programed cell death and its ligand (PD-1/PDL-1) signaling pathway by *T. gondii* [44], in parallel to PD-1 blockers [29]. While PD-1 blockers were effective only in early tumor stages, PD-1/PDL-1 pathway is inhibited by *T. gondii* during both early and chronic infection stages, which potentiates its use in early and late tumor stages [44].

Fibrosis is another tumor prognostic criteria since treatment with chemotherapeutic and immunotherapeutic agents promoted not only necrosis, but also fibrosis [43]. Following chemotherapy, fibrosis enclosing tumor was associated with better pancreatic cancer prognosis [45], as a sort of tissue healing following treatment-induced tumor necrosis [43]. This matches with the present findings, where significantly more fibrosis was noted with all adopted treatments, most prominently with CP/ATV treatment, which justifies the noted difference in tumor weight and volume.

To thoroughly investigate the immune-mediated mechanism, IHC was performed on tumor sections from different groups. Analysis of immune cells, CD8^+^ T and Treg cells, surrounding ESC showed a state of immunosuppressive dominance in ESC control, which coincides with cancer hallmarks [5]. Upon CP treatment, a higher CD8^+^ T cells and lower Treg cells with a higher CD8^+^/Treg cell ratio compared to ESC control were shown surrounding the tumor. This matches with the previously investigated immunomodulatory role of low-dose CP and its influence on Treg cells depletion [15, 16, 17]. Whereas treatment with ATV alone did not influence CD8^+^ T cells surrounding ESC, while inducing a significant Treg cell depletion compared to both ESC control and CP-treated mice with a higher CD8^+^/Treg cell ratio. Moreover, CP/ATV treatment promoted significantly higher CD8^+^ T cells crawling around ESC with Treg cell depletion and a higher CD8^+^/Treg cell ratio, adding more evidence to the speculated antitumoral immunostimulatory synergism between ATV and CP.

Since immune cells infiltrating tumor tissue and the effector T/Treg cells ratio shape and predict cancer outcome [29], we explored the influence of treatments on the immune cells inside the ESC. Both ATV and CP treatments exhibited a significant Treg cell infiltrate depletion, while only ATV significantly induced CD8^+^ T cells infiltration in ESC and increased CD8^+^/Treg ratio inside the tumor. Again, CP/ ATV treatment promoted an antitumoral immunostimulatory synergistic effect with significantly higher CD8^+^ T cells and lower Treg cells with a higher CD8^+^/Treg cell ratio inside ESC compared to either treatment alone.

In fact, Treg cells are a well-established immunosuppressive T cell subtype that enable tolerance to self-antigens by suppressing, in particular, the high affinity antigen-specific cytotoxic T cells and memory cells. However, Treg cells have been linked to immune evasion, and cancer immune-tolerance and progression [5]. Compared to other T lymphocytes, they are especially sensitive to low-dose CP, due to their low levels of intracellular ATP that impairs glutathione production necessary to neutralize CP toxic products. Moreover, low-dose CP has been reported to downregulate the expression of the glucocorticoid-induced TNFR family-related (GITR) gene that is a costimulatory molecule assisting in Treg proliferation. Also, Treg cells have an impaired DNA repair mechanism that cannot resist high-dose CP-mediated killing [17].

Partly as a consequence of CP inhibitory effect on Treg cells, T cell responses to T cell receptor stimulation and the production of tumor antigen-specific T cells are improved [23]. Additionally, reduced Treg by low-dose CP skews T-helper cells from a TH2 to TH1 phenotype, increasing expression of IL-2 gene, which stimulates expansion of memory cytotoxic T lymphocytes [46]. This could explain the currently observed increase in CD8^+^ T cells number and function by low-dose CP that assisted in immunogenic cell death (ICD) of ESC. The ICD is documented by the increased necrosis and fibrosis and the reduction in both ESC weight and volume.

It is to be noted that in this study, a twice dose of CP (50 mg/kg) was adopted two weeks apart. This was based on the reported depletion of Treg cells and increase in CD8^+^ T cells infiltration with a higher CD8^+^/Treg ratio induced by a single dose of 50 mg/kg CP preceding immunotherapy in tumor mice model [16]. Since, Treg cell depletion induced by a single dose of CP is transient and recovery usually follows [15, 16], we adopted a second dose of CP to maintain its immunomodulatory action. Moreover, research conducted using both pathogen and CP as cancer immunomodulators concluded the influence of CP treatment timing in relation to pathogen-derived vaccination as a crucial factor affecting the outcome. If treatments are administered before vaccination, liberation from tumor-associated immune suppression takes place [47]. This justifies our rational use of CP injection one day prior to ATV administration.

An additional cancer criterion is neo-angiogenesis, mediated via VEGF. Scarcity of blood supply flags tumor cell death [5]. Interestingly, VEGF is a dual agent, a proangiogenic factor, and an immunosuppressive promoter. Thus, VEGF level correlates with Treg cell population, while inversely correlates with CD8^+^ T cells within the tumor [48]. This matches with our findings since ESC control showed high VEGF and Treg cell with low CD8^+^ T cell within tumor. Upon CP treatment, significant reduction in tumor VEGF was noted compared to ESC control and ATV-treated mice, which matches with the previous studies [49]. Likewise, ATV treatment significantly reduced VEGF expression in line with studies using different *T. gondii* variants in cancer murine models [50, 51, 52]. Most probably, the noted inhibitory effect of ATV and CP on neovascularization lead to marked tumor hypoxia and avascular necrosis that stunted progressive neoplastic growth.

In this context, ATV can be considered a dual immunotherapeutic agent via a direct immune stimulation by tumor infiltration with CD8^+^ T cells and depletion of the immune-suppressive Treg cells as well as an antiangiogenic action. Apparently, these observed ATV actions would interfere with tumor growth and thus inducing tumor shrinkage by promoting tumor necrosis and fibrosis with subsequent reduction in tumor weight and volume.

The molecular mimicry theory and sharing of glycoprotein antigens between parasites and cancer [7] can be the main tactic by which ATV provoked its observed immunomodulatory antineoplastic activity against ESC. Acknowledging the abundance of proteins linked to N and O glycans in *Toxoplasma* [53], adds a privilege for investigating *Toxoplasma* shared antigens with various cancer cell lines that will not only support its antineoplastic activity, but also pave the way for its involvement in an effective antineoplastic vaccine. Regarding *Toxoplasma* antigen, it appears that its combination with low-dose CP had boosted its immunomodulatory action and assisted in a superior antineoplastic activity. It is well documented that the use of optimized drug combinations against cancer is of optimum benefit not only to synergistically attack different antitumoral pathways, but also to assist in reducing the chemotherapy-induced toxicity and cancer drug resistance. Herein, the addition of ATV to CP enabled a maximum benefit of its low-dose use, thus reducing its potential toxicity that results from the cellular DNA damage induced by its standard anticancer dose [36]. Additionally, cancer immunomodulation helps to induce loads of activated immune cells that are capable of killing tumor cells specifically, thus avoiding major toxicities of traditional chemotherapy. Also, it can overcome cancer drug resistance by enabling a continued tumor immune surveillance [15]. This matches with the previous studies highlighting the synergistic depletion of Treg cells and increased infiltration of CD8^+^ T cells upon combined use of CP and various immunotherapeutic agents [15, 16, 17].

## Conclusion

Autoclaved *Toxoplasma* vaccine provoked a therapeutic antineoplastic potential with an immunostimulatory and antiangiogenic activity by raising CD8^+^/Treg cells ratio inside the tumor superior to cyclophosphamide. Their combined use ameliorated their antineoplastic effectiveness reflecting a synergistic potential with better hepatic profile. Present findings add a privilege of combining parasitic vaccines to chemotherapeutic regimen to boost their antineoplastic effect, improve their tolerability, and lessen their potential resistance, while reducing their dose. Further studies are ongoing for exploration of its prophylactic potency as well as the proposed shared antigen theory between parasites and cancer.

## Data Availability

All raw data will be available upon request.

## Abbreviations

ATV: Autoclaved *Toxoplasma* vaccine
CP: Cyclophosphamide
ESC: Ehrlich solid carcinoma
*ip*: Intraperitoneally
*Toxoplasma gondii*: *T. gondii*
Treg: T regulatory cells
VEGF: Vascular endothelial growth factor
